# PReS-FINAL-2324: PAPA syndrome clinical spectrum and IL-1Β release

**DOI:** 10.1186/1546-0096-11-S2-P314

**Published:** 2013-12-05

**Authors:** A Omenetti, R Caorsi, S Carta, L Delfino, F Penco, C Pastorino, F Schena, A Martini, A Rubartelli, M Gattorno

**Affiliations:** 1 Pediatrics II Unit, G Gaslini IRCCS, Genoa, Italy; 2 University of Genoa, Genoa, Italy; 3 Cell Biology, IRCCS AOU IST-San Martino, Genoa, Italy

## Introduction

Pyogenic sterile Arthritis Pyoderma gangrenosum and Acne (PAPA) syndrome is a rare autosomal dominant inherited autoinflammatory disease caused by mutations of Proline-serine-threonine phosphatase-interacting protein 1 (PSTPIP1). In childhood, the syndrome is featured by recurrent sterile, erosive arthritis, potentially leading to joint destruction. By puberty, cutaneous symptoms become predominant, with recurrent onset of pathergy, abscesses, severe cystic acne, and pyoderma gangrenosum. Typically, both articular and cutaneous outcomes occur following a minor trauma. PSTPIP1 may interact with ASC and caspase-1 but a clear involvement of interleukin (IL)-1β and the role of NLRP3 is still controversial. While anti-IL1 treatment seems to be effective on joint manifestations, IL inhibition does not display the same effectiveness in the management of skin lesions.

## Objectives

To investigate in our PAPA cohort whether IL1β secretion 1) is enhanced, 2) correlates with different PSTPIP1 mutations, clinical manifestations and/or disease activity, and 3) is mediated by NLRP3.

## Methods

Eleven genetically confirmed patients (*N *= 2 children and *N *= 9 adults) carrying different PSTPIP1 mutations (E250K *N *= 1, E256G *N *= 1, E250Q *N *= 9) were analyzed and compared to 31 healthy donors (HD). Four patients had an active disease at the time of sampling while 7 were symptom-free. Nine out of 11 were off-therapy. Peripheral blood primary human monocytes were freshly isolated and studied at baseline and after 3-6-18 hours (h) of LPS-induced *in vitro *activation. Pattern of IL-1β secretion was assessed by ELISA. The involvement of NLRP3 was investigated by in vitro silencing.

## Results

Monocytes isolated from PAPA patients tent to secrete higher levels of IL-1β but this was not consistent in all the patients (p = 0.144). Variability in IL-1β release occurred even in the presence of the same PSTPIP1 variant and it did not parallel disease activity when the whole cohort was taken in consideration. However, IL-1β levels varied according to the clinical picture (p = 0.0197), and it was significantly higher (*P *< 0.05) in patients displaying articular manifestations (*N *= 3) compared to those affected by cutaneous (*N *= 6) or combined (*N *= 2) lesions. In the former, IL-1β secretion was increased in the presence of acute phase reactants elevation and active joint lesions, and treatment with Anakinra resulted in acute phase reactans normalization and symptom-free period. Silencing of of NLRP3 activity consistently inhibited LPS-induced IL-1β release in both PAPA and HD monocytes.

## Conclusion

IL-1β secretion is higher in PAPA patients displaying prevalent articular manifestations. In these patients, IL-1β release correlates with disease activity and it is mediated by NLRP3.

## Disclosure of interest

None declared.

